# Reporting in a health emergency: The roles of Sierra Leonean journalists during the 2014-2015 Ebola outbreak

**DOI:** 10.1371/journal.pntd.0008256

**Published:** 2020-05-21

**Authors:** Maike Winters, Helena Nordenstedt, Helle Mölsted Alvesson

**Affiliations:** Department of Global Public Health, Karolinska Institutet, Stockholm, Sweden; University of Queensland, AUSTRALIA

## Abstract

**Introduction:**

In public health emergencies, local media are important sources of information for the public. Not much is known about the professional and personal roles and experiences of Sierra Leonean journalists during the Ebola virus disease (EVD) outbreak between 2014–2015.

**Methods/Principal findings:**

This qualitative study is based on semi-structured interviews with 13 Sierra Leonean journalists based in urban Freetown and rural Waterloo in February and March 2016. The majority of the journalists worked for radio stations. The mostly male journalists represented national, regional and local radio stations. Transcripts of the interviews were analysed using thematic analysis. The analysis was inspired by previously reported journalist roles and agenda-setting theory, which state that the media filter what is happening, making a few core issues more salient. Sierra Leonean journalists shifted from being sceptical monitors in the beginning of the outbreak, to collaborative instructors towards the end. While they adapted to different roles, journalists struggled with their own fears for the virus, which hampered their work. They indicated that the training they received about Ebola helped them overcome their fear. Being trained gave a sense of security that helped them carry out their jobs. By turning into instructors, journalists stepped away from their journalistic professional detachment–potentially exacerbated by their personal experiences.

**Conclusion/Significance:**

The first months of the outbreak were marked by passive agenda-setting roles of Sierra Leonean journalists. It took several months before the outbreak became a core issue in local media. In health emergencies, efforts should be made to partner with local media to use their platforms for local, trusted journalists and leaders to disseminate public health messages. Whereas this might hamper journalists’ credibility and can be challenging in areas with problematic press freedom, Sierra Leonean journalists experienced the outbreak as a driver of necessary change in their profession.

## Author summary

Local media are important sources of information for the public during a health emergency. However, not much is known about the roles and experiences of local journalists who have to report under extraordinary circumstances. In this qualitative study, we interviewed Sierra Leonean journalists who reported during the 2014–2015 Ebola outbreak. The thematic analysis shows that the journalists adapted different roles over the course of the outbreak. They went from being sceptical about the existence of an outbreak at the start to changing into instructors at the end of the outbreak. Journalists struggled with their own fear for the virus, which they could overcome through targeted training, that helped them identify safe practices of reporting in the field. Our results show that it is possible to effectively partner with local media to disseminate public health messages. This might be challenging in settings with problematic press freedom, but Sierra Leonean journalists actually felt that the outbreak helped improve their practices.

## Introduction

When disaster strikes, people have a direct and strong need for information. Questions arise like: What has happened? Who is affected? How can I protect myself? In many countries, people are advised to turn on the radio or TV to keep informed. No matter the medium, risk communication is one of the vital parts of any disaster, be it an earthquake, a gas leak or a disease outbreak [1].

Ebola virus disease (EVD) was largely unknown in West Africa, before it claimed its first victims in 2014 in Guinea, Liberia and Sierra Leone. Partially due to the slow response in the first months of the outbreak [2], EVD spread rapidly. Over the course of almost two years, more than 28,000 people got infected with EVD, making it the largest outbreak of EVD in history [3]. Of the affected countries, Sierra Leone had the highest number of cases [4]. Risk communication and social mobilization should have informed the public about the disease, giving guidance on how to avoid and how to deal with an infection of EVD [5]. However, it took several months after the start of the outbreak before aligned communication strategies were developed [2,6,7].

In case of a disaster, risk communication guidelines identify local media as an important target group for government and other official institutes to communicate with [1,8]. It is acknowledged that local media have access to local information and can reach their audiences through their medium [1,8]. Therefore, it has been suggested that local journalists can be directly mobilized, or even be commissioned to disseminate public health information [1].

In terms of media coverage, it has been found that disasters can be conceptualized in different phases [9]. In the initial phase of a disaster, there can be speculation in the media, followed by a phase in which journalists commonly try to contextualize the disaster and aim to take on a longer-term perspective [9]. In each of these phases, journalists have been reported to take on different roles [10–12]. For instance, they can be public advocates, shining a light on underrepresented people, a catalyst by drawing attention to budget issues, or a public safety official, giving people warnings and information on risks associated with the disaster [10]. Journalists can turn from independent reporters and watchdogs to public mobilizers, as was seen in the E. coli outbreak in Germany in 2011 and the earthquake in Japan in that same year [11,13]. An often-debated topic is how journalists can balance their core values of independence with disseminating essential public health information–a perceived cooperation with authorities [11,14].

A common assumption among the roles of journalists is that the journalist is an outsider, unaffected by the disaster. However, local journalists can be personally affected if disaster strikes, likely making their lived experiences different than those of the unaffected reporter. A study that looked at experiences of journalists during Typhoon Haiyan in the Philippines, who became victims of the superstorm as well, found that they experienced the disaster in multiple ways: as victims, as journalists, as leaders and as community members [9]. The way the typhoon was experienced in one role, affected the experience of another role [9,15].

To date, not much is known about how local journalists in Sierra Leone experienced the EVD outbreak. In this paper, we therefore aim to understand how Sierra Leonean journalists perceived their professional and personal roles during the EVD outbreak. This study was inspired by the previously described journalist roles as well as agenda-setting theory. The roles of journalists can vary across geography, level of press freedom and situation. Hanitzsch et al. described elementary functions of journalists, such as informational-instructive, critical-monitorial and developmental-educative [16]. Agenda-setting theory states that the media filter what is happening, focusing on a few core issues–making them more salient, while ignoring other stories [17,18]. In doing so, the public can perceive those highlighted issues as more important. Furthermore, these issues can over time become priorities of the public agenda as well [17,18]. Agenda-setting can be seen as a passive or proactive activity, whereby the agenda-setting is a by-product of reporting in the passive stance and is actively sought-after in the active stance [19].

## Methods

### Setting

Radio is the most used medium for the large majority of the public in Sierra Leone. It is estimated that 81% of Sierra Leoneans can access radio, compared to 45% with access to TV and only 13% with access to newspapers [20]. Access to internet from computers or mobile phones is available to 38% of the total population and up to 65% in urban areas [21]. There are currently around 50 functioning radio stations in the country, none of which reaches a national audience. Reaching a national audience can only be done through collaboration among radio stations. In order to do so, the Independent Radio Network (IRN) was set up in 2007 to establish national coverage of the elections. This network was again activated during the EVD outbreak [22].

Sierra Leone has been a media pioneer on the African continent. It was the first West-African country to publish newspapers and to start radio broadcasts [23]. However, libel laws that came into force in 1965 have been used by the Sierra Leonean government to silence journalists [23]. In a survey among Sierra Leonean journalists carried out in 2014–2015, 84% of respondents indicated that media laws were a major external influence on their work [24]. The civil war between 1991 and 2002 brought further damage to the media landscape, with an estimated 70% of media professionals fleeing the country [23]. The return of peace saw a comeback of an increasingly diversified media landscape. However, the libel laws are still in force today, and are still a ground to sue or incarcerate journalists. As a result, press freedom in Sierra Leone has been classified as ‘problematic’ by Reporters without Borders [25].

This study is set in the Western Area in Sierra Leone, inhabited by 1.5 million people out the of the total population of 7 million people. The large majority (around 1 million people) of Western Area inhabitants lives in Freetown, the capital of Sierra Leone. The country ranks amongst the poorest countries on Earth, and its population had a life expectancy of 52 years in 2015 [26]. Since Sierra Leone gained independence from Britain in 1961, two main political parties have governed the country: the All People’s Congress (APC), who were in power during the outbreak and the Sierra Leone Peoples Party (SLPP) who came into power after the 2018 elections [27]. The EVD outbreak started in the Eastern Province–a stronghold of the SLPP, the opposition party at that time. The interviews for this study were carried out in Freetown, where most news outlets have their offices and newsroom, and in Waterloo, a town close to Freetown.

### Study design and participant identification

This is a qualitative study, based on semi-structured interviews with Sierra Leonean journalists and stakeholders of risk communication, and was reported according to COREQ guidelines [28]. Journalists were identified through purposive sampling, based on the predefined criteria that the journalist was currently working in a news outlet and had reported during the EVD outbreak. Mostly radio journalists were sampled, which reflected the media landscape of Sierra Leone, and was in line with the finding that radio was the most important source of information about EVD during the outbreak [29].

Semi-structured interviews were conducted with 13 journalists in 12 interviews in February and March 2016, a few weeks after the last two EVD cases were reported in Sierra Leone. The majority of journalists (11 out of 13) were radio journalists, 1 worked for a newspaper and 1 wrote for an online news site (see Table 1). Apart from 2 journalists working for the same radio station in Freetown, all journalists came from different radio stations or media outlets. The sample was predominantly male and the majority worked in Freetown. Three journalists worked in the neighbouring town Waterloo. Many interviewees identified themselves in various different media roles at the same time, such as journalist, producer, presenter and editor. All of the interviewees have in common that they reported during the Ebola outbreak, they are therefore all identified as journalists in this study.

**Table 1:** Demographics of the journalists.

Demographic	Sample
Location	10 in Freetown (urban) 3 in Waterloo (rural)
Age in years (mean, range)	37 (29–49)
Sex	10 men 3 women
Education	4 high school 9 university
Medium	11 radio 1 newspaper 1 online news site
Ebola training access	3 direct access to training & trainers themselves 10 receiving training
Ebola stakeholders	1 international non-governmental organization 1 Sierra Leonean media expert 1 Ministry of Health and Sanitation representative in Sierra Leone 1 international expert deployed to the Ministry of Health in Liberia

Ebola training access (Table 1) refers to the level of access the journalists had to information and training about Ebola and was assessed by the authors after the interview was conducted. Three journalists in Freetown co-organized trainings for journalists during the outbreak and were directly involved in the creation of the messages disseminated during the outbreak. The other 10 journalists were mostly disseminating these messages further. Apart from the interviews with the 13 journalists, four additional interviews with Ebola stakeholders were carried out. These additional interviews were instrumental to better understand the media landscape, to get reflections on the role of the Ministry of Health during the outbreak, and to gain insight into the perspectives of an international organization.

### Data collection

The interviews were carried out through the lens of the chronological epidemic curve: initially there were few Ebola cases, in remote areas of Sierra Leone. When the virus spread to the capital, there was an exponential increase in cases for a few weeks in November 2014, before the outbreak was under control and the number of new patients started to go down until the last cases were managed in January 2016. The interview guide was informed by quantitative data analysis looking at the association between information sources and Ebola-specific knowledge and behaviour in Sierra Leone [30]. The study found associations between information channels like radio and knowledge, protective behaviour and misconceptions about the virus [30]. The semi-structured interviews followed an interview guide, containing questions about journalists’ awareness of Ebola before the outbreak, as well as questions about experiences, information sources, knowledge and behaviour during the outbreak. The sequencing of the questions depended on the flow of information with each participant. Each interview took place at the workplace of the journalists; in radio studios and newsrooms and were conducted in English. MW was in Freetown for two months in 2016 to carry out the interviews with the journalists as part of a Master’s thesis in Public Health Epidemiology, and currently conducts doctoral research in Freetown.

### Data analysis

The interviews were transcribed verbatim and were analysed in NVivo 11, using thematic analysis as a guidance in the analysis [31]. Initial coding was limited to a few transcripts. Agreement on the coding scheme was reached through multiple discussions with co-authors, before being applied to all transcripts. Through comparing and contrasting between journalists, the analysis became more latent, with a detailed analysis of the themes. Comparing and contrasting was done to understand potential differences between rural and urban-based journalists as well as between journalists with various levels of access to training about Ebola. Themes focused on roles of the journalists, but were not limited to the roles. The analysis was an iterative process, which involved going back to literature for further understanding of certain topics and using this new knowledge to interpret the data. Agenda-setting theory helped to place the emerging roles on a continuum of professional journalist behaviours, as described by McCombs [19]. Four professional behaviours are described on this continuum: professional detachment, targeted involvement, boosterism and proactive agenda setting. Agenda setting is considered a passive byproduct of the first three behaviours on this continuum [19]. During the course of the analysis, peer examinations [32] were carried out with co-authors and an academic Sierra Leonean peer, who provided feedback on emerging themes using his own experience of working during the outbreak. The emerging themes were examined using the main roles and agendas that comprise agenda-setting theory and journalist roles [9,10,15,31].

### Ethical considerations

All interviewees read and signed an informed consent form. The Sierra Leone Research and Scientific Review Committee granted ethical permission for this study retrospectively in August 2018. The study was also approved by the Ethical Review Board in Stockholm, Sweden (dnr 2018/1276-31).

## Results

**Fig 1:**
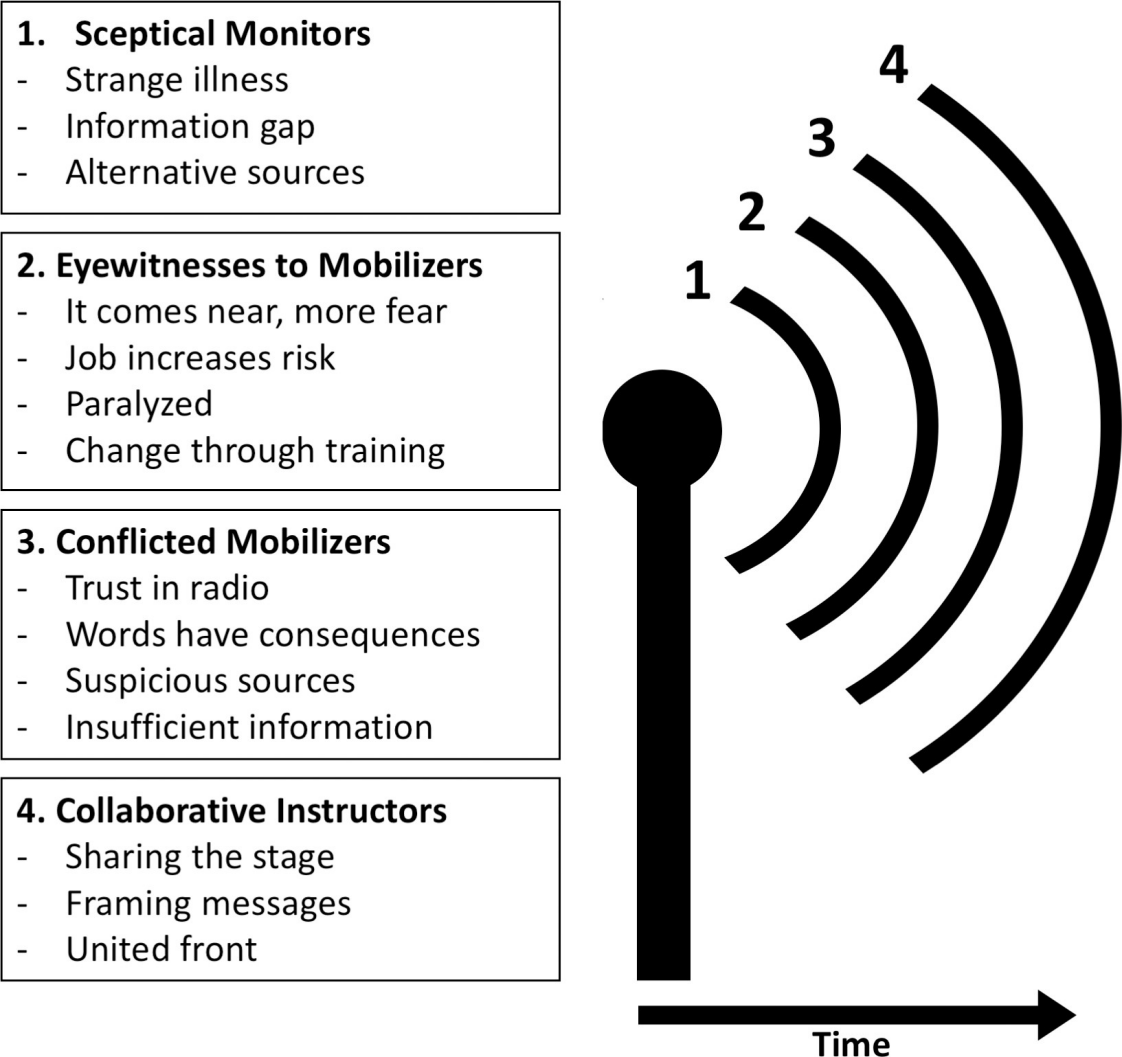
Themes and categories.

Four themes focusing on roles and subsequent categories were identified in the data. The themes follow a chronological order, in line with how the journalists recounted their experiences. Themes and categories are visually represented in Fig 1 and are discussed and illustrated with quotes below.

### Journalists as sceptical monitors

Journalists indicated that the first months of the outbreak were characterized by a mix of uncertainty, confusion and ignoring the existence of an outbreak. Together with a lack of forthcoming information from official sources, the situation gave rise to several political storylines around the outbreak, according to the journalists. The first EVD cases in Sierra Leone were confined to the Eastern Province, a rural area where the political opposition at that time had a large following [27]. In Freetown, a governmental stronghold, there was talk about a new disease, but it wasn’t taken very seriously, according to the journalists.

‘…a strange illness had arrived, it transferred to contacts and it is killing people fast. But then, we didn’t take it seriously. Because it was confined to a far part of the country. The attitude was ‘it’s not my business’, to some extent that was also the attitude of the government.’ (Journalist 6)

Given the geopolitical divide in the country, journalists recalled that initially there were different explanations about the nature and the cause of EVD.

‘There was this perception among people that Ebola was a political tool, meant to wipe out the population of people where it all started. Because at that time, the census program was very close…So the views that people held… that this was a strategy by the government to reduce their populations, so that when the census program comes, they will be limited in number and that has a ripple effect on their development. Because the census influences the number of constituencies and seats they should have in parliament and councils.’ (Journalist 10)

The journalists themselves were not convinced about the existence or the nature of the disease either and were rather passively monitoring what was happening:

*‘Ebola is not Ebola*, *it is a political fight*. *So while they* [the public] *are spending much time on this wrong information* [political explanations about the outbreak], *Ebola was moving*. *By the time I came to realize that yes there is a sickness now*, *it had overcome so many districts*.*’ (Journalist 13)*

All journalists said that they were looking for information around the new mysterious disease. The Ministry of Health and Sanitation (MoHS) in Sierra Leone did not provide the clarity they needed. They therefore looked for other sources that could help them make sense of the situation.

*‘Some of the major challenges we had here*, *was how to get to them* [MoHS]. *Just to explain the Ebola thing*. *It took time*. *We had to go to other communities to interview health experts*, *asking them questions*, *asking to give information*.*’ (Journalist 3)*

Getting increasingly convinced about the existence of an outbreak, journalists conveyed that another source of information was through eyewitness accounts of people in the affected areas.

‘*We used people as agents*, *to talk to the people… We used the people who came back from the provinces as agents on the radio to tell the community what they had seen*.*’ (Journalist 3)*

### From eyewitnesses & victims to educators & public mobilizers

Journalists discussed that while different storylines about EVD emerged, the virus managed to spread from the Eastern Province to the entire country. Now that the outbreak affected the Western Area, the province of both Freetown and Waterloo, the journalists’ perception of risk changed, as the disease was suddenly near, fatal and potentially catastrophic [33]. Especially in Waterloo, journalists were first-hand witnesses of the outbreak.

*‘That was in November 2014*. *Because from August*, *September*, *that was the hell of time of Ebola in Sierra Leone*. *We* [Sierra Leone] *registered up to 200 cases across the country*. *In Western Rural*, *they were calling us Ebola manufacturers*.*’ (Journalist 1)*

The journalists were not only reporters during the EVD outbreak. They were also citizens of an epidemic-ridden country, meaning that their professional jobs as reporters were also influenced by their own direct experiences of the outbreak. For most of the journalists in the sample, the disease came very close–some had family members contracting the diseases, others lost friends to EVD. With the journalists experiencing the outbreak as eyewitnesses and victims themselves, their scepticism made place for fear. Being eyewitnesses and victims to the unfolding disaster, the journalists conveyed that due to the nature of their jobs they were potentially more exposed to the virus than the public, which made them fear for their own and their family’s safety.

‘Initially we were so scared, even of recording. We thought what if the person was talking and the saliva just came on your hand? … How do we hold the microphone?’ (Journalist 4)

‘It was, so scary. Ebola is so bad. Actually, I was more concerned, not about myself, because if you are a journalist then and you are going to the field, you know there was a risk. And if you went out you must be ready for that risk. But I was more concerned about my family. My wife was just pregnant, I kept thinking whether I was going to see my child. That was something I really thought about. So it was really scary, really.’ (Journalist 8)

The perceived risk of personal consequences of their jobs also hampered all journalists in their work. They said that they still experienced a lack of necessary information from official sources, such as the MoHS. The journalists in rural Waterloo recalled that it was especially difficult for them to get information, as they perceived that officials would normally only talk to the bigger news outlets in Freetown.

‘During the early period of the outbreak, as journalists we were actually waiting for someone to explain to us, having somebody engage with the media, telling this is Ebola. And when it affects you, this is how you feel. It was not done in the early moment of the outbreak.’ (Journalist 11)

Some of the journalists in Freetown who had access to government officials and international organizations managed to get health experts to come to their newsrooms to explain the ins and outs of EVD.

*‘At one point*, *the CDC* [The US Centers for Disease Control and Prevention] *organized a media orientation for journalists to educate us on what Ebola was all about*. *The signs and symptoms of Ebola*, *what you need to do*, *what are the best practices for us to stop transmission of the disease*.*’ (Journalist 12)*

The main effect these training sessions seemed to have was to reduce fear and to improve practical knowledge of how to report during the outbreak. Subsequently, more training sessions were organized, initiated by media stations in Freetown, to reach journalists in other corners of the country. Two of the journalists in this study gave training to fellow journalists, five journalists explicitly mentioned that they received training.

‘Once we had that knowledge, we had the power to feel the fear, but to confront the enemy anyways.’ (Journalist 4)

### Journalists as conflicted mobilizers

Journalists perceive themselves to be highly trusted in their communities. Because of their perceived status, most journalists said that they realized that their words had consequences–especially in an outbreak situation like EVD.

‘This is a country where people say ‘I heard it on the radio, so it must be true.’ So you had to be careful about what you said. And at least once or twice, there were potential problems. In Kenema, somebody was brought on the radio, he was a former health worker I think. That person started a rumour on the radio, and that created a big problem in Kenema. They took offence on the hospital, the hospital was attacked, vandalized. It was all a conspiracy, this person said, and the journalist didn’t challenge that, you see. Which is why it was very important to train the journalists properly.’ (Journalist 4)

With the knowledge they gained from the training, the journalists indicated that they started actively disseminating information that was increasingly in line with what official sources were conveying to the public. Several journalists said that their audiences were suspicious of their link with the government. The balancing act between giving the right information and keeping the trust of their audiences was perceived to be difficult by some journalists, as explained here:

‘Some people said, you guys are talking too much, that means that the government gives you a lot of money, that’s why you are talking.’ (Journalist 1)

Another problem the journalists discussed was that while they received more information from official sources, they still felt it was not enough to educate their audiences and lacked practical tips on how to handle Ebola. Information they gave raised more questions, which they felt they could not answer.

‘We educated the people a lot, but the information that we passed at that time was not that enough. Sometimes it was contradictory. Because you tell people don’t come near the sick, but who is going to take care of the sick? We never knew the methods of how to take care of the sick, somebody that has contracted Ebola, and all of those things.’ (Journalist 1)

The mobilizing role of the journalists became more prominent and evolved into new emerging role: instructors.

### Journalists as collaborative instructors

As the outbreak continued, all radio journalists described that they became partners of the response, producing radio programs together that were broadcast simultaneously on many different radio stations across the country. Journalists conveyed that they started sharing their stage with outbreak experts and local leaders, believing that trusted voices in different segments of communities could use their platform to reach their followers.

*‘People* [journalists] *host swabbers* [*i*.*e*. those who take samples from corpses to determine the cause of death], *burial team members*, *doctors*, *nurses at ETU’s*, *traditional healers and leaders*. *Healers talking to other healers and ask them to stop their practices for a while*. *Religious leaders asking people not to touch dead bodies until after swabbers had come and confirmed it was negative*. *Through that medium*, *each category of people had been targeted*.*’ (Journalist 11)*

Sharing the stage also meant that journalists had to take a step back, which made them reflect on their position.

‘Through you information can pass. But don’t think you are the expert. Don’t think you know more than other people.’ (Journalist 4)

‘We are there as an intermediary to pass the message to and from. So, what we are getting, is what we are disseminating.’ (Journalist 13)

As the role of journalists changed, the content of their messages also shifted. The message ‘Ebola has no cure’ that was disseminated in the first months, changed to highlight the chance to survive. Survivors were brought on air, to tell their experiences of treatment centres and encourage people to go there when symptoms of EVD appeared. Messages contained mostly practical information according to the journalists, instructing what people should do in certain scenarios.

‘What we were doing was getting medical experts to come and tell us, tell members of the public what the disease was all about. How one can become infected with the virus and what you have to do once you start to experience the signs and symptoms of Ebola. Report early for treatment.’ (Journalist 12)

The roles of journalists did not only change in terms of taking a step back and sharing the stage. The nature of their job transformed from being independent reporters to collaborative instructors, aiming to give their audiences practical advice around Ebola.

‘Before Ebola, our editorial policy was just to give our views and information. We don’t tell people what to do. We give our views and information and allow people to make their own decisions. When Ebola came, we realized it should be a different type of information dissemination. In fact, it was communication and not information. And by communication it means telling people what to do and what not to do.’ (Journalist 6)

Being part of the response confirmed to journalists the power of their platforms. Most journalists indicated that especially radio was essential in the outbreak response.

‘The radio played a pivotal role in disseminating the information on Ebola. Had it not been the radio, then I wonder what it would have been at this point in time.’ (Journalist 12)

Looking back, most journalists reflected that reporting during the outbreak improved the quality of their reporting. The questions from their audiences made them follow-up on stories.

‘Before the coming in of Ebola, there are some journalists who are just interested in attending a press conference, without doing a follow-up on that particular story. But with Ebola, we were having questions from the public…. Ebola in a way helped us to complete stories.’ (Journalist 11)

## Discussion

Informing audiences facing a potentially fatal outbreak is a priority in combatting the disease. Understanding the roles of those already disseminating information provides valuable insights that could be used in future health emergencies. The Sierra Leonean journalists in this study conveyed how their roles changed during the course of the EVD outbreak. Placing the roles in the agenda-setting framework, it can be discerned that roles in the first months of the outbreak, specifically the sceptical monitor, can be classified as rather passive agenda-setting roles; the agenda-setting (i.e. making a topic more salient) was a by-product of their news production [17–19]. As time went on and the outbreak intensified, journalists became public mobilizers–indicating a proactive stance towards agenda-setting [19]. Whereas agenda-setting theory limits the agenda-setting roles to passive and proactive agenda-setting [19], it can be argued that journalists in our study went beyond proactive agenda-setting in their role as collaborative instructors. It was no longer about making certain topics more salient; the outbreak was already the only major topic at that time [6]. Instead, it was about health promotion and social and behaviour change communication [34,35]. More attention was instead placed on framing the messages (also called ‘second-level agenda-setting’ [36,37]), highlighting the chances of survival from EVD for instance.

The journalistic professional detachment was side-stepped when the journalists became instructors. This finding is contrary to previously reported journalist roles in disaster settings, where journalists tried to maintain independence despite disseminating public health messages [10–14]. Journalistic independence is a construct that is ascribed different importance in different parts of the world [38]. In a study that surveyed journalists in 67 different countries, Sierra Leonean journalists placed less value on being a detached observer than journalists from high-income countries [39]. This might be partially due to the problematic state of press freedom in Sierra Leone [25]. It can also be explained by the finding that Sierra Leonean journalists placed more importance on roles such as educating their audience, supporting national development and advocating for social change than their peers in high-income countries [24,39]. It could be presumed that these influences and role perceptions might have influenced the journalists’ willingness to give up their professional detachment to become instructors.

Being personally affected by the EVD outbreak might also have played a role in the willingness of the Sierra Leonean journalists to circumvent their professional detachment. A wide range of personal, professional and situational factors influences how journalists might cope with a disaster situation [9,33,40,41]. It can be speculated that being personally affected by an unfolding disaster increased the perceptions of risk and the perceived need of the Sierra Leonean journalists to warn and inform the public. Their position in the media also gave them access to information about the virus ahead of the public–a privileged position, especially in an outbreak situation [19]. Knowledge can have an empowering effect [42]; this was also seen in our sample of journalists, where their newly acquired knowledge was a turning point. It reduced their levels of fear for the virus and they felt that they could carry out their jobs again–with the necessary precautionary measures at work and at home.

In health emergencies such as the ongoing Ebola outbreak in the Democratic Republic of the Congo, efforts should be made to leverage local media as platforms for locally trusted journalists and leaders to disseminate public health messages. This might pose a dilemma as journalists might be deemed credible by the public because they are perceived to be independent from the government. Professional detachment in their reporting about other topics should therefore be encouraged. Still, adverse effects in terms of loss of credibility of the journalists cannot be excluded. Interestingly, in our study we have seen that the EVD outbreak was experienced as a driver of change in the media in Sierra Leone. As one interviewee said: ‘Ebola in a way helped us complete stories.’

### Strengths and limitations

The Sierra Leonean journalists in our study form a unique group, as they experienced the outbreak as professional journalists and as citizens.

The interviews were carried out by MW, who has a background in journalism. This was an advantage in terms of understanding the nature of the job of a journalist, potentially enhancing rapport with the interviewees. At the same time, this could also have influenced the phrasing of questions and the probing during the interviews. Apart from one interview, the journalists were open and provided rich accounts of their experiences. It has been argued that interviewing the interviewers might pose a difficulty in itself [43]. Journalists might be aware of misrepresentation of the gathered data, and could therefore be sceptical about being interviewed [43]. Even with these precautions in mind, the journalists did not seem to hold back during the interviews. The peer examinations we did with a Sierra Leonean national with working experience in the EVD outbreak added rigor to the analysis.

Given their profession, journalists are likely to have an inherent belief in the power of information. They might have overstated the importance of information in the interviews, potentially also due to social desirability. Lastly, this study did not include the media output the journalists produced. We can therefore not check whether the statements of the journalists were in line with what they produced during the outbreak.

The information power of the sample was evaluated according to the guidelines developed by Malterud [44]. Limitations of this sample include the underrepresentation of journalists from rural areas. Furthermore, journalists did not seem to be strong in self-reflection and might have overestimated their roles in the outbreak. Taken together, the information power of the sample is deemed to be sufficient for the purpose of this study.

### Conclusion

This study shows that Sierra Leonean journalists took on different roles as the EVD outbreak developed, even roles that are considered to be outside of traditional journalism. Journalists stepped away from their professional detachment to become instructors for the official response. When the virus spread to the Western Area, the perceived lack of forthcoming information from official sources about the virus and the course of the outbreak instilled fear in the journalists, hampering their work. Being trained and gaining knowledge was one of the turning points for the journalists. In health emergencies, efforts should be made to partner with local media for the dissemination of public health messages.
